# The Prognostic Value of the Detection of Microbial Translocation in the Blood of Colorectal Cancer Patients

**DOI:** 10.3390/cancers12041058

**Published:** 2020-04-24

**Authors:** Ippokratis Messaritakis, Konstantinos Vogiatzoglou, Konstantina Tsantaki, Agapi Ntretaki, Maria Sfakianaki, Asimina Koulouridi, John Tsiaoussis, Dimitrios Mavroudis, John Souglakos

**Affiliations:** 1 Laboratory of Translational Oncology, Medical School, University of Crete, 70013 Heraklion, Crete, Greece; vogiatzogloukwstas@gmail.com (K.V.); kon.tsantou@gmail.com (K.T.); agapis97@gmail.com (A.N.); mimasf19@gmail.com (M.S.); asi_minakoulouridi@yahoo.com (A.K.); mavroudis@uoc.gr (D.M.); johnsougl@gmail.com (J.S.); 2 Laboratory of Anatomy, Medical School, University of Crete, 70013 Heraklion, Crete, Greece; tsiaoussis@uoc.gr; 3 Department of Medical Oncology, University General Hospital of Heraklion, 70013 Heraklion, Crete, Greece

**Keywords:** dysbiosis, microbial translocation, bacterial translocation, colorectal cancer, toll-like receptors, vitamin D receptors

## Abstract

Dysbiosis has been associated with various diseases and is of major health importance. Dysbiosis leads to microbial translocation, which is the passage of microorganisms, their fragments, or their metabolites from the intestinal lumen into the blood circulation and other sites. The aim of the study was to determine whether microbial translocation occurs in stage II/III-IV colorectal cancer (CRC) patients. The aim was also to evaluate the usefulness of blood PCR for diagnosis of such translocation and correlate the presence of toll-like receptor/vitamin D receptor (TLR/VDR) gene polymorphisms with microbial DNA fragments detected in the blood of CRC patients. Three hundred and ninety-seven CRC patients enrolled in the study. Peripheral blood DNA was analyzed using PCR for the amplification of genomic DNA encoding 16S rRNA, the *β*-galactosidase gene of *Escherichia coli*, glutamine synthase gene of *Bacteroides fragilis*, and *5*.8S rRNA of *Candida albicans*. Significantly higher rates of all microbial fragments, but *E. coli*, detected were observed in the CRC patients (*p* < 0.001); such detection of all four microbial fragments was also significantly associated with the metastatic disease (*p* < 0.001), leading to shorter survival rates (*p* < 0.001). Tumor location in the right colon also significantly correlated with shorter survival (*p* = 0.016). Individuals with homozygous mutant alleles of TLR/VDR polymorphisms had significantly higher detection rates of microbial DNA fragments. The detection of microbial DNA fragments in CRC patients highlighted the role of these microbes in cancer development, progression, and patients’ survival.

## 1. Introduction

Intestinal dysbiosis, termed as variation in composition and diversity of the gut microbiota, has been associated with multiple metabolic and immune conditions and is considered of major public health importance [1]. The mechanisms linking the gut microbiota to human health are mostly unknown, but healthier individuals often harbor greater microbial diversity [2]. Dysbiosis can lead to the passage of viable microbes from the intestinal lumen through the mesenteric lymph nodes and other sites, known as microbial translocation. However, the concept of microbial translocation does not limit on microorganisms’ translocation only but also on their products or fragments, such as endotoxins, peptidoglycan, lipopeptides, and nucleic acids [3]. Apart from dysbiosis, gut barrier dysfunction and increased permeability (also known as a leaky gut syndrome) may arise due to several health conditions, including autism, diabetes, obesity, depression, organ disorders, inflammatory bowel disease, and cancer [4,5,6,7,8].

Microbial translocation has been previously determined in animals [9,10]. Examples include the quantitation of viable microbial colonies isolated from tissues distant to the gut or the intestinal administration of radioisotope-labeled microbes (or their products), followed by measurement of radioactivity in the tissues [9,10]. However, blood microbial cultures are often negative, and the repetitive administration of radioisotope-labeled microbes in the human gut is not an easy task. Molecular techniques have also been developed for the detection of microbial fragments in various clinical specimens [11,12,13,14,15].

The primary goal for the present study was to determine whether microbial translocation occurs in colorectal cancer (CRC) patients and to evaluate the usefulness of PCR for the diagnosis of such translocation in the blood of these patients. CRC is one of the most common cancers leading to death, worldwide, representing 9% of all malignancies [16]. Its development has been widely associated with genetic mutations, diet, inflammatory conditions, and dysbiosis [17]. The secondary objective of the study was to determine the correlation of the presence of, previously investigated by our group, toll-like receptors (TLRs) [18] and vitamin D receptors (VDRs) polymorphisms [19] with the detection of microbial DNA fragments in the blood of CRC patients.

## 2. Results

### 2.1. Patients’ Characteristics

Table 1 and Data S1 summarize the demographics of all CRC patients enrolled in the study. In brief, the median age of the patients was 65 years (range 18–88 years), 246 (62.0%) were males, 202 (50.9%) were of adjuvant setting, 279 (70.3%) had tumor location on colon/sigmoid, 74 (25.1%) had tumors of the right colon, 372 (93.7%) had a good performance status according to the eastern cooperative oncology group (PS-ECOG) (0–1), 205 (47.4%) had tumors of high grade, and 104 (42.4%) of those tested for KRAS (Kirsten rat sarcoma viral oncogene homolog) were mutant (Table 1 and Data S1).

### 2.2. Microbial DNA Detection in the Blood of CRC Patients and Controls

DNA coding for 16S rRNA was detected in 256 (64.5%) CRC patients and 5 (15.6%) healthy controls (*p* < 0.001); *β*-galactosidase gene of *E. coli* was detected in 104 (26.2%) CRC patients and 5 (15.6%) controls (*p* = 0.186); glutamine synthase gene of *B. fragilis* was detected in 220 (55.4%) CRC patients and in 0 (0%) controls (*p* < 0.001); whereas, DNA coding for 5.8S rRNA of *C. albicans* was detected in 230 (57.9%) CRC patients and in 0 (0%) controls (*p* < 0.001) (Table 2 and Data S1).

**Figure 1:**
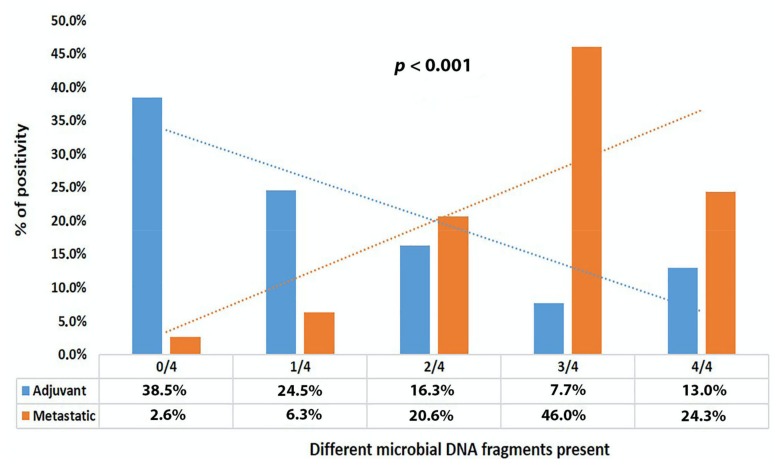
Rate of microbial DNA fragments among colorectal cancer (CRC) stages.

Then, patients were grouped according to their disease stage. A higher detection rate of DNA fragments coding for 16S rRNA, *β*-galactosidase of *E. coli*, glutamine synthase of *B. fragilis*, and 5.8S rRNA was detected in patients with metastatic disease compared to adjuvant patients (88.4% vs. 42.8%, *p* < 0.001; 31.7% vs. 21.2%, *p* = 0.017; 82.0% vs. 31.2%, *p* < 0.001; 81.0% vs. 37.0%, *p* < 0.001, respectively) (Table 2 and Data S1). Moreover, the correlation of the two groups of patients showed a detection of mainly three or four (46% or 24.3%) different microbial DNA fragments per metastatic patient and a detection of mainly none or one (38.5% or 24.5%) per adjuvant patient (*p* < 0.001) (Figure 1 and Data S1).

### 2.3. Association of Microbial DNA Detection and Clinical Outcome

**Figure 2:**
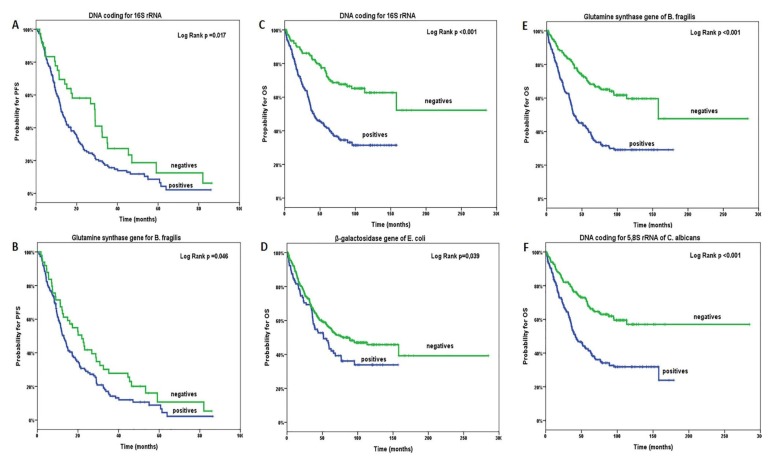
Progression-free survival (PFS; **A**–**D**) and overall survival (OS; **E**–**H**) of patients, according to the detection of microbial DNA fragments.

Following their treatment, 36 (13.8%) adjuvant and 176 (85.9%) metastatic patients presented disease progression (Data S1). For stage II/III patients, the median disease free survival (DFS) and Overall survival (OS) was 19 months (95% CI: 15.5–22.5) and 65 months (95% CI: 59.1–250.9), respectively; whereas, for stage IV patients, the median progression free survival (PFS) was 8 months (95% CI: 7.1–8.9) and 31 months (95% CI: 25.2–36.8), respectively. For the total number of enrolled patients, the median PFS was 14.1 months (95% CI: 11.5–16.7), and the median OS was 65.8 months (95% CI: 46.7–84.9) (Data S1). According to the detection of the microbial DNA, a significantly shorter PFS was observed in patients with detectable microbial DNA fragments coding for 16S rRNA and glutamine synthase gene of *B. fragilis* (*p* = 0.017 and *p* = 0.046, respectively) (Figure 2A,B). No significantly shorter PFS was observed in patients with detectable microbial DNA fragments coding for *β*-galactosidase of *E. coli* and 5.8S rRNA of *C. albicans* (Figure 2C,D). Moreover, a significantly shorter OS was observed in patients with detectable microbial DNA fragments coding for 16S rRNA, *β*-galactosidase of *E. coli*, glutamine synthase gene of *B. fragilis*, and 5.8S rRNA of *C. albicans* (*p* < 0.001, *p* = 0.039, *p* < 0.001, and *p* < 0.001, respectively) (Figure 2E–H). Additionally, no statistical significance between the survival outcome and microbial DNA presence was presented in either stage II/III or stage IV CRC patients, alone.

### 2.4. Association of Tumor Location and Clinical Outcome

**Figure 3:**
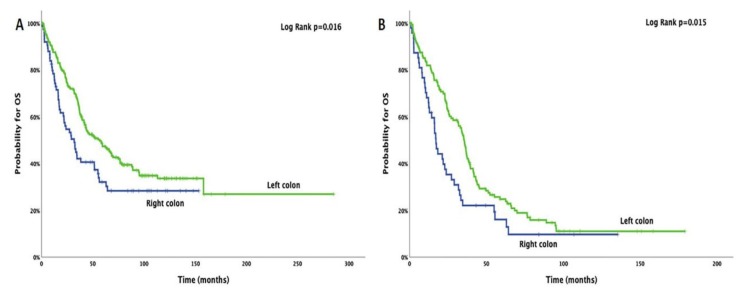
Overall survival of the whole group of patients (**A**) and the metastatic setting (**B**) according to the tumor-sidedness.

As described in Table 1 and Data S1, 221 (74.9%) and 74 (25.1%) patients had tumor location in the left (rectum, sigmoid, or ascending colon) and right colon (transverse, descending colon, or cecum), respectively. Patients with right side tumor location presented a significantly shorter OS than those with a left side tumor location, and this was observed both for the whole group of patients (median 36.8 vs. 56.9 months; 95% CI: 21.0–42.6 vs. 41.7–72.1; *p* = 0.016) and the metastatic group (median 17.1 vs. 35.5 months; 95% CI: 14.3–19.9 vs. 32.4–39.6; *p* = 0.015) (Figure 3A,B). Among patients with right-sided tumors, 73%, 31.1%, 67.6%, and 68.9% had detectable 16S rRNA, *E. coli*, *B. fragilis*, and *C. candida* DNA fragments, respectively, in their blood; whereas, 70.1%, 26.7%, 58.8%, and 61.1% of the patients with left-sided tumors had detectable 16S rRNA, *E. coli*, *B. fragilis*, and *C. candida* DNA fragments, respectively (Data S1). Moreover, in patients with detectable microbial DNA, sidedness of the tumor location also presented a significant difference in their survival. Patients with detectable 16S rRNA, *B. fragilis*, or *C. candida* DNA fragments and right-sided tumors presented a significantly shorter OS than those with a left-sided tumors, and this was the case both for the whole group of patients (*p* = 0.045, *p* = 0.046, and *p* = 0.040, respectively) and the metastatic group (*p* = 0.012, *p* = 0.013, and *p* = 0.025, respectively) (Data S1).

### 2.5. Correlation of Microbial DNA Presence and TLR and VDR Polymorphisms

**Figure 4:**
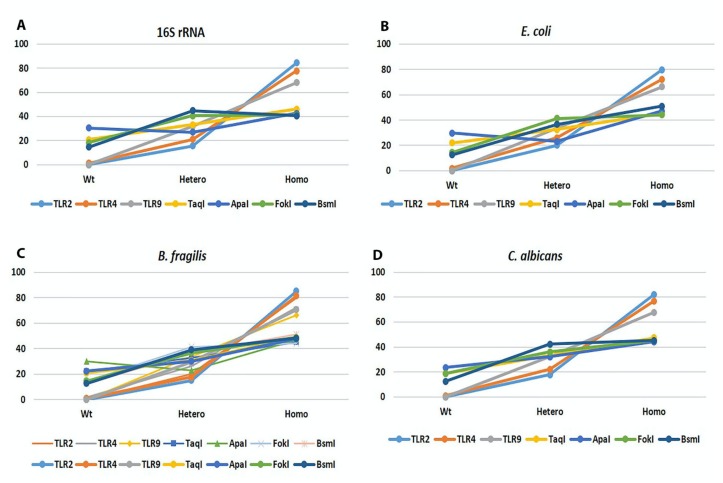
Association of microbial DNA fragments detected in the blood of CRC patients and TLR/VDR DNA polymorphisms. Association of DNA polymorphisms with (**A**) DNA coding for 16S rRNA, (**B**) β-galactosidase gene of *E. coli*, (**C**) Glutamine synthase gene of *B. fragilis* and (**D**) DNA coding for 5.8S rRNA of *C. albicans*.

TLR2 (−196 to −174 bp), TLR4 (Asp299Gly and Thr399Ile), and TLR9 (T1237C and T1486C) polymorphisms and *Taq*I, *Apa*I, *Fok*I, and *Bsm*I polymorphisms of the VDR gene have been described previously [18,19], and their correlation with the presence of microbial DNA in the blood of CRC patients was investigated (Table 3). A significant coexistence was observed between the detection of all four different microbial DNA fragments with all different VDR and TLR polymorphisms. More specifically, homozygotes for the mutant allele of every single polymorphism analyzed had a higher detection rate of all four microbial DNA fragments (Table 3 and Figure 4A–D).

### 2.6. Univariate and Multivariate Analysis

Univariate analysis revealed that the disease stage (IV vs. II/III), the detection of microbial DNA encoding for 16S rRNA, glutamine synthase of *B. fragilis*, and 5.8S rRNA were significantly associated with a shorter PFS; and tumor location (right vs. left colon), disease stage (IV vs. II/III), the detection of microbial DNA encoding for 16S rRNA, *β*-galactosidase of *E. coli*, glutamine synthase of *B. fragilis*, and 5.8S rRNA were significantly associated with shorter OS (Table 4). In multivariate analysis, adjusting for these factors, only disease stage (IV vs. II/III) and the detection of microbial DNA encoding for 16S rRNA emerged as independent factors associated with decreased PFS (HR: 1.7, 95% CI: 1.0–2.7, *p* = 0.037; HR: 1.5, 95% CI: 1.0–2.2, *p* = 0.029); whereas, disease stage (IV vs. II/III) and both the detection of microbial DNA encoding for 16S rRNA and glutamine synthase of *B. fragilis* emerged as independent factors associated with decreased OS (HR: 6.4, 95% CI: 4.3–9.3, *p* < 0.001; HR: 2.0, 95% CI: 1.4–2.9, *p* < 0.001; HR: 1.9, 95%CI: 1.3–2.8, *p* < 0.001) (Table 4).

## 3. Discussion

The human body is not only made up of body cells but also of microbes, most of which are living in the gut. The commensal microbiota provides a significant contribution to maintaining homeostasis and regulating nutrition, metabolism, immunity, and inflammation [20,21,22,23]. However, gut dysbiosis may lead to microbial translocation from the intestine to the blood circulation, leading to carcinogenesis if not eliminated by the defense mechanisms [24,25,26,27,28,29,30,31]. The identification of microbial translocation in the blood by the standard blood cultures has often been hard due to either low microbial load or the use of antibiotics [32,33]. Other complicated and not so sensitive methods include the repetitive administration of radioisotope-labeled microbes in the human gut [9,10]. Therefore, there has been an urgent need for the development of a highly sensitive surrogate marker for the diagnosis of microbial translocation. To this end, various research groups have attempted to develop PCR-based methods for the detection of microbial fragments in the blood of patients undergoing surgeries, with chronic myalgias, liver transplantations, Crohn’s diseases, etc. [10,11,34,35,36]. However, to our knowledge, little progress has been made, and small cohorts have been enrolled in the detection of microbial translocation in the blood of cancer patients. 

In the current study, we aimed to use a PCR-based method for the detection of microbial (bacterial and/or fungal) DNA in the blood of patients with adjuvant or metastatic CRC. In a previous study, Rodriguez-Laiz et al. evaluated the gut bacterial DNA translocation in the blood of 100 patients undergoing liver transplantation [35]. Bacterial DNA was detected in the blood of 26% and 34% of patients undergoing liver transplantation and after one month, respectively. Interestingly, such translocation contributed to systemic inflammation, but no impact on clinical outcome was reported [35]. In another study, the authors analyzed blood samples from 40 surgical patients for the detection of 16S rRNA, *β*-galactosidase of *E. coli*, and glutamine synthase of *B. fragilis* [11]. Of those enrolled, all transplant patients receiving muromomab-CD3 (OKT3) had detectable microbial DNA in their blood; whereas, 64% of the critically ill patients had detectable microbial DNA in their blood, with positive blood cultures in only 14% of them [11]. Li Y. et al. aimed to detect bacterial DNA in the blood of 107 patients with Crohn’s disease undergoing abdominal surgery [34]. Bacterial DNA was detected in 27.1% of these patients, and such detection was significantly associated with a longer postoperative hospital stay and increased postoperative adverse outcomes [34]. Moreover, Ota A et al. analyzed 39 blood samples from various cancer patients at different time points during chemotherapy [37]. Their findings supported the detection of bacterial translocation in cancer patients, regardless of the chemotherapy administration, thus leading to febrile neutropenia or other treatment-associated infections [37]. Ono S et al. analyzed 52 blood samples from surgical patients for the diagnosis of gut microbial translocation [10]. Their analysis revealed a bacterial DNA presence in septic patients and those with hepatic lobectomy; whereas, *C. albicans* DNA presence was detected in septic patients and those with esophagectomy. Both bacterial and fungal DNA was detected in patients receiving chemotherapy for advanced colon cancer. Again, none of the patients had a positive blood culture [10]. Finally, Lescut D et al. enrolled 20 CRC and 20 non-CRC patients under surgery [38]. Bacterial translocation was significantly higher in CRC patients (65% vs. 30%, *p* < 0.005), thus highlighting the need for prophylactic antibiotic treatment in CRC surgery [38]. Our results were in agreement with the above-mentioned studies. A significantly higher detection rate of DNA fragments coding for 16S rRNA, glutamine synthase gene of *B. fragilis*, and 5.8S rRNA of *C. albicans* was detected in the pool of 397 CRC patients compared to healthy subjects, thus highlighting the role of these microbes in CRC development. At this point, it has to be mentioned that a limitation of the current study was the relatively small number of the control group. However, in order to overcome such a limitation, the age distribution of the control subjects was close to that of patients with the same percentage per decade (with the exception of patients older than 70 years age) and equal in gender and region of residence. Moreover, a higher detection rate of DNA fragments coding for 16S rRNA, *β*-galactosidase of *E. coli*, glutamine synthase of *B. fragilis*, and 5.8S rRNA was detected in patients with metastatic disease compared to adjuvant patients, thus highlighting the role of these microbes in CRC development. Patients with detectable microbial DNA in their blood also presented shorter DFS, PFS, and OS, which was again in agreement with previously mentioned studies.

The clinical outcome of the patients enrolled in our study was also associated with the anatomical location of the tumor. It was observed that patients with right-sided tumors presented a significantly decreased OS compared to left-sided tumors. This phenomenon has also been described previously [39,40,41,42]. Baran B et al. reported that treatment response differed among the left and right-sided tumors. The authors mentioned that right-sided tumors usually respond only to immunotherapies due to their high antigenic load [39]; however, as addressed by others, despite no benefit in OS has been demonstrated, available data can endorse the use of an anti-epidermal growth factor receptor (anti-EGFR) treatment in right-sided RAS (retrovirus-associated DNA sequence) wild-type advanced CRC in tumor shrinkage [43]. Moreover, a meta-analysis concluded that in the right-sided advanced CRC, chemotherapy plus bevacizumab was a treatment option [44,45]. Moreover, the higher antigenic load described in the right-sided tumors is more likely to be due to the defective mismatch repair mechanism because of the high frequency of mutations, thus increasing the possibility of immune recognition [46,47]. Lim DR et al. described data from 414 patients under curative resection for right or left-sided colon cancer, suggesting that patients with left-sided tumors had a better survival outcome than those with right-sided tumors, especially in stage III patients [41]. Finally, Ulanja MB et al., in a retrospective analysis of 163,980 patients with colon cancer, concluded that tumor sidedness might affect presentation and survival rates at different stages of the disease. They reported that patients with left-sided tumors presented better overall survival rates in stages I, III/IV; whereas, patients with right-sided tumors presented better survival rates only in stage II disease [42]. The results from our study and the studies mentioned above could be explained by the findings of Dejea CM et al. The authors examined 36 tissues from the right (15 CRC and 4 adenomas) and left-sided tumors (15 CRC and 2 adenomas) and demonstrated the association of bacterial aggregates (biofilms) in the colon with CRC. Such biofilms are present in the vast majority of right-sided tumors, leading to increased bacterial tissue invasion, microsatellite instability, hypermethylation, hypermutation, and BRAF^V600E^ mutations, thus increasing the risk for CRC development five-fold in patients with biofilms [40,48].

As described previously by our group and others, TLR and VDR polymorphisms have been significantly associated with cancer development, progression, and, therefore, patients’ clinical outcome [18,19,49,50]. Linked to the fact that *TLR* and the *VDR* genes play an important role in the prevention and elimination of infections, their mutations result in the impaired activation of these receptors, decreased response to microbial particles, and impaired homeostasis, thus leading to carcinogenesis and disease progression [51,52,53,54,55,56]. In the present study, we found a significant correlation between all microbial DNA fragments present in the blood of CRC patients and polymorphisms of the *TLR* and *VDR* genes. This significant correlation confirmed the fact that such polymorphisms negatively influence the host immunity against microbial translocation and persistence in the blood circulation, thus leading to cancer development and progression.

## 4. Materials and Methods

### 4.1. Enrollment of Patients

During the period February 2005 to October 2013, 397 patients aged > 18 years old with newly diagnosed and histologically documented colorectal cancer (CRC) were enrolled in the study. All patients were treated at the Department of Medical Oncology, University Hospital of Heraklion.

### 4.2. Ethics Approval and Consent to Participate

The study has been approved by the Ethics Committee/Institutional review board of the University Hospital of Heraklion Number (7302/19-8-2009), and all patients signed written informed consent for their participation. All procedures performed were in accordance with the ethical standards of the institutional and/or national research committee and with the 1964 Helsinki declaration and its later amendments or comparable ethical standards.

### 4.3. Blood Samples and Genomic DNA Extraction

Peripheral blood (5 mL in EDTA) was obtained from all CRC patients (*n* = 397) and healthy individuals (*n* = 32) as control subjects. Peripheral blood from all CRC patients was collected just before the initiation of any adjuvant or first-line treatment. DNA extraction from the whole blood was performed using the QIAamp DNA Blood Mini kit (QIAGEN, Hilden, Germany) following the manufacturer’s instructions. DNA was quantified using the NanoDrop ND-1000 v3.3 (Thermo Fisher Scientific, Wilmington, DE, USA)

### 4.4. Microbial DNA Amplification by PCR

All primer sequences, PCR conditions, and size of amplicons (bp) for each gene target are summarized in Table 5. In brief, four oligonucleotide primer pairs were used to detect genomic DNA encoding 16S rRNA found in all Gram-positive, Gram-negative bacteria, and mycobacteria [57,58,59]; *β*-galactosidase gene of most *Escherichia coli* but not many other gram-negative bacteria [60]; glutamine synthase gene of *Bacteroides fragilis* [61], and 5.8S rRNA found in *Candida albicans* [62]. Moreover, a pair of primers was also included as a reference gene to detect genomic DNA encoding human glyceraldehyde phospho-dehydrogenase in all patients and controls (Table 5).

### 4.5. Study Design and Statistics

The present single-institution study was a retrospective study, aiming to investigate the detection of microbial DNA fragments in the blood of CRC patients before their treatment initiation. Progression-free survival (PFS) and overall survival (OS) were calculated, as previously described [18], and all experiments were performed blindly to the clinical data. Statistical analysis was performed using the SPSS v. 20 software (IBM Corp. Armonk, NY, USA), based on contingency tables, including hazard ratios (HR) and 95% CI calculations, as previously described [18]. Statistical significance was set at *p* = 0.05.

## 5. Conclusions

In conclusion, our study provided strong evidence of the presence of microbial DNA in the blood of CRC patients. Our findings highlighted the significant role of the detection of DNA fragments coding for 16S rRNA found in all Gram+/Gram-/mycobacteria, *β*-galactosidase of *E. coli*, glutamine synthase of *B. fragilis*, and 5.8S rRNA of *C. albicans*, in the development and progression of CRC, thus affecting the clinical outcome of such patients. Clearly, this increased risk in CRC patients should be taken into consideration by the clinicians when planning any surgical interventions or during chemotherapy.

## Figures and Tables

**Table 1:** Patients’ characteristics.

Characteristics	Frequency (*n* = 397)	%
Age (range)	65 (18–88)	
18–50	52	13.1
51–70	221	55.7
>70	124	31.2
Gender		
Male	246	62
Female	151	38
Stage		
IIA–IIIC	202	50.9
IV	195	49.1
Location		
Left colon	221	74.9
Right colon	74	25.1
Site		
Colon/sigmoid	279	70.3
Rectum	118	29.7
PS (ECOG)		
0–1	372	93.7
≥2	25	6.3
Surgery		
Yes	347	87.4
No	50	12.6
Adjuvant treatment		
Yes	230	57.9
No	167	42.1
First-line treatment		
Yes	223	56.2
No	174	46.8
Grade		
High	205	47.4
Low	232	52.6
KRAS		
Mutant	104	42.4
Wild type	141	57.6
Not Determined	152	

**Table 2:** Association of the presence of microbial DNA between patients and control groups and among patients.

Gene Target	Detection	Patients	Healthy Blood Donors	*p*-Value	Stage II/III	Stage IV	*p*-Value
DNA coding for 16S rRNA	Positive	256 (64.5%)	5 (15.6%)	<0.001	89 (42.8%)	167 (88.4%)	<0.001
	Negative	141 (35.5%)	27 (84.4%)		119 (57.2%)	22 (11.6%)	
β-galactosidase gene of *E. coli*	Positive	104 (26.2%)	5 (15.6%)	0.186	44 (21.2%)	60 (31.7%)	0.017
	Negative	293 (73.8%)	27 (84.4%)		164 (78.8%)	129 (68.3%)	
Glutamine synthase gene of *B. fragilis*	Positive	220 (55.4%)	0 (0%)	<0.001	65 (31.2%)	155 (82.0%)	<0.001
	Negative	177 (44.6%)	32 (100%)		143 (68.8%)	34 (18.0%)	
DNA coding for 5.8S rRNA of *C. albicans*	Positive	230 (57.9%)	0 (0%)	<0.001	77 (37.0%)	153 (81.0%)	<0.001
	Negative	167 (42.1%)	32 (100%)		131 (63.0%)	36 (19.0%)	

**Table 3:** Association of the presence of microbial DNA and toll-like receptor (TLR) and vitamin D receptor (VDR) gene mutant alleles (the value against each category is *p*-value).

	TLR			VDR			
Gene Target	TLR2	TLR4	TLR9	Taq*I*	Apa*I*	Fok*I*	Bsm*I*
DNA coding for 16S rRNA	<0.001	<0.001	<0.001	<0.001	<0.001	<0.001	<0.001
β-galactosidase gene of *E. coli*	0.365	0.823	0.233	0.028	0.001	<0.001	<0.001
Glutamine synthase gene of *B. fragilis*	<0.001	<0.001	<0.001	<0.001	<0.001	<0.001	<0.001
DNA coding for 5.8S rRNA of *C. albicans*	0.002	0.001	0.002	<0.001	<0.001	<0.001	<0.001

**Table 4:** Univariate and multivariate Cox regression analysis for progression free (PFS) and overall (OS) survival.

Parameters	Univariate Analysis				Multivariate Analysis			
	PFS		OS		PFS		OS	
	HR (95% CI)	*p*-Value	HR (95% CI)	*p*-Value	HR (95% CI)	*p*-Value	HR (95% CI)	*p*-Value
Tumor location (Right vs. Left Colon)	1.1 (0.8–1.6)	0.441	1.4 (1.0–1.8)	0.05	-	-	1.3 (0.9–1.7)	0.118
Age	1.1 (0.8–1.3)	0.483	1.0 (0.99–1.02)	0.103	-	-	-	-
Stage (IV vs. II/III)	2.0 (1.3–3.2)	0.002	7.3 (5.2–10.2)	<0.001	1.7 (1.0–2.7)	0.037	6.4 (4.3–9.3)	<0.001
16S rRNA	1.6 (1.1–2.4)	0.01	2.7 (1.9–3.8)	<0.001	1.5 (1.0–2.2)	0.029	2.0 (1.4–2.9)	<0.001
β-galactosidase of *E. coli*	1.1 (0.8–1.4)	0.738	1.4 (1.0–1.9)	0.04	-	-	0.9 (0.6–1.2)	0.282
Glutamine synthase of *B. fragilis*	1.5 (1.1–2.1)	0.019	2.6 (1.9–3.5)	<0.001	1.4 (1.0–1.9)	0.061	1.9 (1.3–2.8)	<0.001
5.8S rRNA of *C. albicans*	1.1 (0.8–1.4)	0.058	2.3 (1.7–3.1)	<0.001	1.1 (0.8–1.6)	0.564	1.2 (0.8–1.7)	0.378

**Table 5:** PCR primers designed to amplify microbial and human DNA fragments.

Target Gene	Primer	Sequence	PCR Conditions	Fragment Size
GAPDH	F	5′-TCT CCA GAA CAT CAT CCT G-3′	Denaturation at 95 °C for 5 min. Then, samples were exposed to 35 cycles of denaturing (95 °C, 1 min), annealing (60 °C, 1 min), and extension (72 °C, 1 min), followed by a final extension step at 72 °C for 10 min	324 bp
	R	5′-GAG CTT GAC AAA GTG GTC GT-3′		
16S ribosomal RNA for Gram+ and Gram- bacteria	F	5′-AGT TTG ATC CTG GCT CAG-3′		798 bp
	R	5′-GGA CTA CCA GGG TAT CTA AT-3′		
β-galactosidase to detect *E. coli*	F	5′-CTT GCC TGG TTT CCG GCA CCA GAA-3′		762 bp
	R	5′-AAC CAC CGC ACG ATA GAG ATT CGG G-3′		
lutamine synthase to detect *B. fragilis*	F	5′-ACT CTT TGT ATC CCG ACG ACG ATT-3′		581 bp
	R	5′-GAG GTT GAT GCC TGT ATA TCG GT-3′		
45.8S ribosomal RNA to detect *C. albicans*	F	5′-TCC GTA GGT GAA CCT TGC GG-3′	Denaturation at 95 °C for 5 min. Then, samples were exposed to 35 cycles of denaturing (94 °C, 1 min), annealing (56 °C, 2 min), and extension (72 °C, 2 min), followed by a final extension step at 72 °C for 10 min	550 bp
	R	5′-TCC TCC GCT TAT TGA TAT GC-3′		

## Supplementary Materials

The following are available online at https://www.mdpi.com/2072-6694/12/4/1058/s1, Data S1: Raw data material.
